# CT-derived body composition and differential association with age, TNM stage and systemic inflammation in patients with colon cancer

**DOI:** 10.1038/s41598-024-65871-y

**Published:** 2024-07-08

**Authors:** Allan M. Golder, Michael Ferguson, Paul McMillan, David Mansouri, Paul G. Horgan, Campbell S. Roxburgh, Ross D. Dolan, Josh McGovern, Donald C. McMillan

**Affiliations:** Academic Unit of Surgery, University of Glasgow, Level 2, New Lister Building, Glasgow Royal Infirmary, Glasgow, G31 2ER UK

**Keywords:** Age, Sarcopenia, Body composition, CT, Cancer, Colorectal, Cancer imaging, Biomarkers, Gastrointestinal cancer, Colorectal cancer, Cancer epidemiology, Cancer metabolism

## Abstract

Low skeletal muscle index/density (SMI/SMD) is prevalent in cancer, adversely prognostic and associated with tumour stage and the systemic inflammatory response (SIR). Age and SMI/SMD has not been widely studied. The present study analyses the association between age and SMI/SMD after adjustment for other clinicopathological factors. Patients undergoing resectional surgery for TNM Stage I-III disease within the West of Scotland between 2011 and 2014 were identified. A single CT slice was obtained from each patients staging CT scan. SMI and SMD were stratified normal/abnormal. The SIR was stratified using Systemic Inflammatory Grade (SIG). When stratified by age (< 50/50s/60s/70s/80+), 39%/38%/48%/62%/74% and 27%/48%/64%/82%/92% of patients had a low SMI and SMD respectively (both p < 0.001). Older age (OR 1.47, p < 0.001), female sex (OR 1.32, p = 0.032), lower socioeconomic deprivation (OR 1.15, p = 0.004), higher ASA (OR 1.30, p = 0.019), emergency presentation (OR 1.82, p = 0.003), lower BMI (OR 0.67, p < 0.002) and higher SIG (OR 1.23, p < 0.001) were independently associated with low SMI. Older age (OR 2.28, p < 0.001), female sex (OR 1.38, p = 0.038), higher ASA (OR 1.92, p < 0.001), emergency presentation (OR 1.71, p = 0.023), and higher SIG (OR 1.37, p < 0.001) were independently associated with lower SMD. Tumour factors were not independently associated with either SMI/SMD. Age was a major factor associated with low SMI/SMD in patients with colon cancer. Therefore, in these patients it is likely that this represents largely constitutional body composition as opposed to being a disease mediated effect. Adjustment for age is required when considering the cancer mediated effect on SMI/SMD in patients with colon cancer.

## Introduction

Sarcopenia, coined by Irwin Rosenbeg in 1989^1^ for an age-related reduction in lean muscle mass, until recently, had no widely accepted definition or diagnostic criteria. In 2009 (and subsequently updated in 2019^2^, the Sarcopenia Working Group defined sarcopenia as “progressive and generalized loss of skeletal muscle mass and strength” that may be primary (aging) or secondary (including disease, nutrition or activity related). Low muscle mass combined with impairment of muscle function, has been recognized for decades however was only defined as an independent disease entity within the ICD-10 classification in 2016^3^. This has corresponded with an increased research output around body composition, in particular low skeletal muscle mass/strength (sarcopenia) and low skeletal muscle density within a number of areas of clinical practice, including cancer.

Multiple modalities of stratifying body composition including dual energy x-ray absorptiometry (DXA), computed tomography (CT) and magnetic resonance imaging (MRI) techniques have been described^4^. Within oncology, the use of CT imaging for stratification of skeletal muscle mass/density dominates the literature, due to the widespread availability of CT imaging routinely carried out for cancer staging^5^. Predominantly, a single CT slice obtained at the level of the third lumbar vertebrae is analyzed with muscle area and density being stratified as previously described by Martin and colleagues^6^. Low skeletal muscle mass (based on CT image analysis) is typically used as a surrogate for sarcopenia.

In patients with cancer, low skeletal muscle mass has been reported to be highly prevalent. A recent meta-analysis by Surov and colleagues^7^ including approximately 80,000 patients with solid organ tumours reported an overall prevalence of low skeletal muscle mass of 35% (28% in colorectal cancer) as measured using a number of body composition methodologies. Including only CT-derived body composition analysis, a recent systematic review by McGovern and colleagues^8^, in approximately 40,000 patients with solid organ tumours, reported an overall prevalence of low skeletal muscle index of 43% (46% in colorectal cancer) and an overall prevalence of low skeletal muscle density of 49% (46% in colorectal cancer). Low skeletal muscle mass and density has been widely reported to be associated with a range of adverse short-term and long-term outcomes within clinical practice^9–14^ including increased chemotherapy toxicity^15,16^.

Cachexia, recently defined according to the GLIM criteria^17^ requires the presence of at least one of three phenotypical criteria (weight loss, low BMI or low muscle mass) and one of two etiological criteria (reduced food intake/absorption or increased disease burden/inflammation) and has traditionally been associated with advanced cancer. However, it is now recognized that low skeletal muscle mass and density is highly prevalent in patients undergoing resectional surgery with curative intent for non-metastatic disease. Some studies have shown an association between more advanced tumour stage and low skeletal muscle index/density^18^ however this association is not universal^19,20^. Furthermore, studies have shown that after adjustment for the systemic inflammatory response, the association between TNM Stage and skeletal muscle index/density is less clear suggesting that systemic inflammation represents a significant confounding factor^21–23^.

It would appear likely that low skeletal muscle index and density as stratified on CT imaging may be widespread within a normal, healthy population however the investigation of this has understandably been constrained due to ethical issues around irradiation of healthy volunteers and the difficulties associated with obtaining an unbiased control group. Indeed, the loss of high-quality lean muscle has been strongly linked to aging with a decline in muscle strength being observed from around the age of 40^24,25^. A previous publication by Martin and colleagues of 2100 patients undergoing resectional surgery for TNM I-IV colorectal cancer did report an association between increasing age and lower skeletal muscle index and skeletal muscle attenuation however did not include patients over the age of 80^14,25^ and did not consider the preoperative systemic inflammatory response. Otherwise, the association between body composition and age has not been widely examined in patients with colorectal cancer. As defined by the GLIM criteria, cachexia is not the result of older age therefore further investigation into the association between age and body composition is required with consideration given to the need to stratify body composition by age to determine a disease-mediated effect on muscle mass and density.

The present study aims to examine the association between the age at diagnosis of patients undergoing resectional surgery with curative intent for TNM I-III colon cancer and association with CT-derived body composition parameters, in particular skeletal muscle mass and density after adjustment for other potential confounding factors including tumour stage and the systemic inflammatory response. It seems likely that age represents a major confounding factor in skeletal muscle mass and density that, unlike sex or BMI is not accounted for using established methods of body composition analysis.

## Methods

All patients diagnosed with, and undergoing curative surgery for, TNM Stage I-III colon cancer within the West of Scotland between January 2011 and December 2014 were identified as previously described^26^. This included four health boards (Ayrshire and Arran, Forth Valley, Lanarkshire and Greater Glasgow and Clyde) and approximately half of the population of Scotland. These patients receive treatment in line with national guidelines and are followed up for a period of 3–5 years. Previous studies have reported these datasets to be of high quality^27,28^. During the time period studied, a bowel screening programme was in existence utilizing guiaic acid faecal occult blood testing (gFOBT). Individuals aged 50–75 were invited automatically on a biennial basis. Individuals aged under 50 were not eligible to participate in screening and individuals aged over 75, although not routinely invited, were eligible to request screening.

Patients undergoing curative surgery for either an elective or emergency diagnosis of TNM I-III colon cancer were included. Those patients with TNM Stage IV disease, rectal (including rectosigmoid) tumours, patients with macroscopically involved margins (R2 resections) and those patients undergoing local/palliative procedures were excluded.

Tumours were staged using the TNM classification system. Socioeconomic status has been classified using the Scottish Index of Multiple Deprivation (SIMD)^29^. The preoperative systemic inflammatory response was classified using the Systemic Inflammatory Grade (SIG)^26^. This SIG utilizes widely available laboratory tests (neutrophil and lymphocyte count, albumin and C-reactive protein) and combines the neutrophil–lymphocyte ratio and modified Glasgow Prognostic Score. The neutrophil–lymphocyte ratio is stratified < 3/3–5/ > 5 and assigned 0/1/2 respectively. The modified Glasgow Prognostic Score is stratified 0 (CRP ≤ 10), 1 (CRP > 10 and albumin ≥ 35) or 2 (CRP ≥ 10 and albumin < 35). These are added cumulatively to give an overall SIG 0–4. Comorbidity was measured according to the American Society of Anaesthesiology (ASA) grade, recorded prospectively.

Patient age at diagnosis has been stratified < 50/50–59/60–69/70–79/80+. Tumours proximal to the splenic flexure were considered right sided and tumours at or distal to the splenic flexure were considered left sided. Preoperative blood results were regarded as the most recent set of preoperative blood results, in elective patients within 1 month prior to surgery and in emergency patients from admission to hospital.

### Anthropometric measurements

A single CT image was obtained at the level of the third lumbar vertebra from the routine staging scan carried out at time of cancer diagnosis taken in the portal venous phase. Scans with significant movement artefact or a missing region of interest were excluded. Images were analysed using a freeware program—NIH ImageJ Version 1.52 (National Institute of Health, USA, https://imagej.net/ij/). Previous research by Mourtzakis and colleagues^30^ compared DXA scanning to CT imaging for both adipose and non-adipose tissue and found the results to be comparable when using Slice-O-Matic software (https://www.tomovision.com/products/sliceomatic.html). Previous studies have shown comparable results between Slice-O-Matic and ImageJ. Indeed, ImageJ appears to be more accurate when compared to Slice-O-Matic as the latter over-estimates muscle mass^31^.

Standard HU ranges were used to define adipose tissue (− 190 to − 30) and skeletal muscle (− 29 to + 150). Measurements were made of subcutaneous fat area, visceral fat area, skeletal muscle area (all cm^2^) and skeletal muscle density (mean HU). Subcutaneous fat area and skeletal muscle area were normalized for height^2^ to create subcutaneous fat and skeletal muscle indices (SFI and SMI respectively, cm^2^/m^2^). Stratification of anthropometric measurements as normal/abnormal was carried out for SFI, VFA, SMI and SMD as previously described by Ebadi^32^, Doyle^33^, Martin^6^ and Martin^6^ respectively (Table 1). Body composition analysis was carried out by AG, MF and PM using methods consistent with the above studies. Inter-relater reliability was assessed within a training cohort of 40 patient images by interclass correlation coefficient (> 0.996).

**Table 1:** Stratification of body composition.

High subcutaneous fat index		
Males	Ebadi^32^	> 50 cm^2^m^2^
Females		> 42 cm^2^m^2^
High visceral fat area		
Males	Doyle^33^	VFA > 160
Females		VFA > 80
Low skeletal muscle index		
Males, BMI ≤ 25	Martin^6^	SMI < 43
Males, MBI > 25		SMI < 53
Females, BMI ≤ 25		SMI < 41
Females, BMI > 25		SMI < 41
Low skeletal muscle density		
BMI < 25	Martin^6^	< 41 HU
BMI ≥ 25		< 33 HU

Body composition analysis was carried out in accordance with validated and established methods. Ethical approval was granted for this project from the Public Benefit and Privacy Panel (PBPP) for Health and Social Care (NHS Scotland). PBPP is a governance structure of the Scottish Government that scrutinizes and authorizes information governance requests. As such, and given the population level nature of this study, individual informed consent was not required. PBPP approval waived the need for informed consent.

### Statistical analysis

The association between clinicopathological characteristics (including markers of CT-derived body composition) and age (categorical) was examined using the Chi-squared test. Two tailed p values have been used throughout with a p < 0.05 considered significant. The association between markers of body composition and other clinicopathological factors has been examined using binary logistic regression to calculate odds ratios (ORs) and 95% confidence intervals (95% CIs). Variables with a p-value of < 0.1 on univariate analysis were entered into a backwards conditional multivariate model.

### Ethics approval and consent to participate

This has been approved by the Public Benefit and Privacy Panel (PBPP) for Scotland. Given the population scale of this data individual patient consent is not required.

## Results

**Figure 1:**
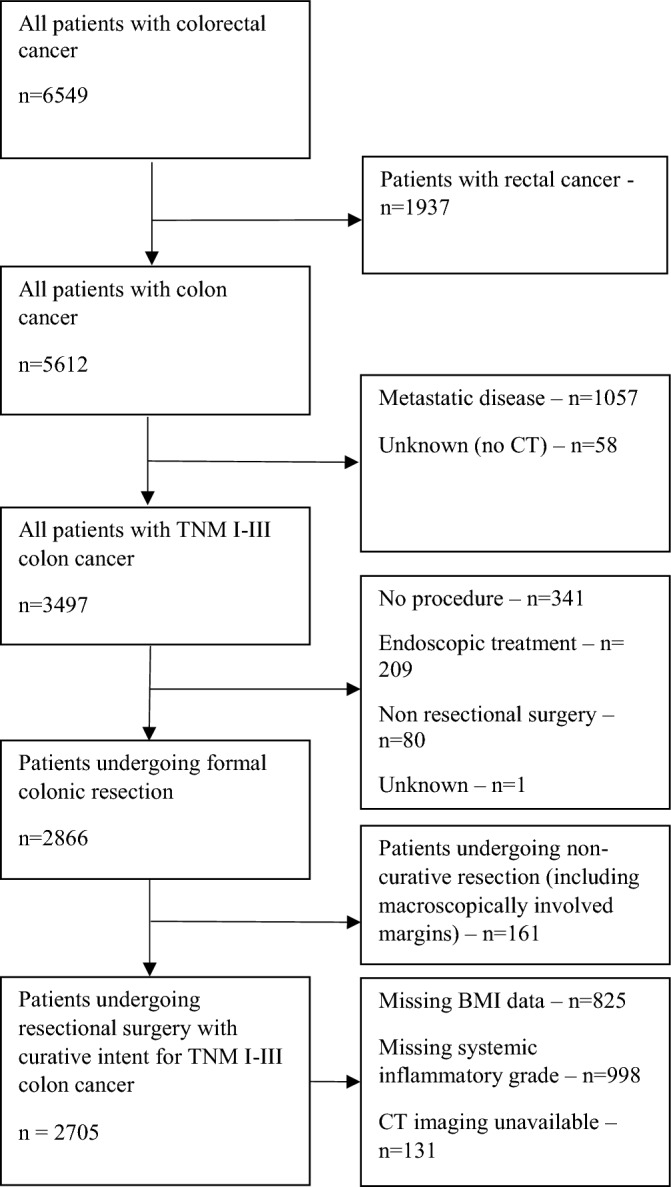
Flow diagram of patient inclusion.

Patient characteristics are shown in Tables 2, 3. 2705 patients were identified undergoing resectional surgery with curative intent for TNM I-III colon cancer within the study period as shown in Fig. 1. 5%/14%/29%/34%/18% of patients were aged < 50/50–59/60–69/70–79/80+ respectively. The median patient age at time of diagnosis was 70 (interquartile range 14). The majority of patients were male (52%) and presented electively (84%) with node negative disease (64%). 80% and 76% of patients had a high subcutaneous fat index and visceral fat area respectively. 54% and 69% of patients had a low skeletal muscle index and skeletal muscle density respectively. Of all patients included in this study: 1850 were invited to participate in the national bowel cancer screening programme, 1124 returned a screening test and 675 patients were diagnosed with cancer through participation in screening.

**Table 2:** Association between age and tumour/host factors.

	Total	< 50	50s	60s	70s	80+	p*
Sex	2705	124 (5%)	368 (14%)	793 (29%)	930 (34%)	490 (18%)	0.007
Male	1403 (52%)	53 (43%)	187 (51%)	444 (56%)	488 (53%)	231 (47%)	
Female	1302 (48%)	71 (57%)	181 (49%)	349 (44%)	442 (48%)	259 (53%)	
SIMD	2705	124 (5%)	368 (14%)	793 (29%)	930 (34%)	490 (18%)	0.091
1	755 (28%)	34 (27%)	113 (31%)	223 (28%)	275 (30%)	110 (22%)	
2	580 (21%)	22 (18%)	82 (22%)	182 (23%)	192 (21%)	102 (21%)	
3	489 (18%)	22 (18%)	51 (14%)	130 (16%)	176 (19%)	110 (22%)	
4	425 (16%)	23 (19%)	59 (16%)	123 (16%)	146 (16%)	74 (15%)	
5	456 (17%)	23 (19%)	63 (17%)	135 (17%)	141 (15%)	94 (19%)	
ASA	2582	116 (5%)	353 (14%)	760 (29%)	890 (35%)	463 (18%)	< 0.001
1	258 (10%)	36 (31%)	70 (20%)	93 (12%)	49 (6%)	10 (2%)	
2	1410 (55%)	62 (53%)	213 (60%)	450 (59%)	490 (55%)	195 (42%)	
3	810 (31%)	17 (15%)	65 (18%)	200 (26%)	317 (36%)	211 (46%)	
4	102 (4%)	1 (1%)	5 (1%)	16 (2%)	34 (4%)	46 (10%)	
5	2 (< 1%)	0	0	1 (< 1%)	0	1 (< 1%)	
Mode of presentation	2705	124 (5%)	368 (14%)	793 (29%)	930 (34%)	490 (18%)	< 0.001
Elective	2263 (84%)	85 (69%)	312 (85%)	681 (86%)	818 (88%)	367 (75%)	
Emergency	442 (16%)	39 (32%)	56 (15%)	112 (14%)	112 (12%)	123 (25%)	
Tumour side	2684	123 (5%)	362 (14%)	784 (29%)	928 (35%)	487 (18%)	< 0.001
Right	1442 (54%)	51 (42%)	162 (45%)	375 (48%)	529 (57%)	325 (67%)	
Left	1242 (46%)	72 (59%)	200 (55%)	409 (52%)	399 (43%)	162 (33%)	
TNM stage	2705	124 (5%)	368 (14%)	793 (29%)	930 (34%)	490 (18%)	< 0.001
I	556 (21%)	13 (11%)	102 (28%)	193 (24%)	184 (20%)	64 (13%)	
II	1161 (43%)	47 (38%)	132 (36%)	310 (39%)	417 (45%)	255 (52%)	
III	988 (37%)	64 (52%)	134 (36%)	290 (37%)	329 (35%)	171 (35%)	
Differentiation	2695	122 (5%)	367 (14%)	787 (29%)	929 (35%)	490 (18%)	0.007
Mod-well	2218 (82%)	95 (78%)	315 (86%)	669 (85%)	754 (81%)	385 (79%)	
Poor	477 (18%)	27 (22%)	52 (14%)	118 (15%)	175 (19%)	105 (21%)	
EMVI	2657	123 (5%)	361 (14%)	775 (29%)	915 (34%)	483 (18%)	0.017
Negative	1538 (58%)	58 (47%)	220 (61%)	459 (59%)	542 (59%)	259 (54%)	
Positive	1119 (42%)	65 (53%)	141 (39%)	316 (41%)	373 (41%)	224 (46%)	
SIG	1707	89 (5%)	219 (13%)	470 (28%)	589 (35%)	340 (20%)	< 0.001
0	571 (34%)	22 (25%)	88 (40%)	180 (38%)	203 (35%)	78 (23%)	
1	410 (24%)	19 (21%)	56 (26%)	120 (26%)	144 (24%)	71 (21%)	
2	327 (19%)	20 (23%)	39 (18%)	82 (17%)	112 (19%)	74 (22%)	
3	214 (13%)	19 (21%)	29 (13%)	50 (11%)	58 (10%)	58 (17%)	
4	185 (11%)	9 (10%)	7 (3%)	38 (8%)	72 (12%)	59 (17%)	
Screening diagnosis	2706	124 (5%)	368 (14%)	793 (29%)	930 (34%)	490 (18%)	< 0.001
No	2036 (75%)	124 (100%)	219 (60%)	493 (62%)	709 (76%)	490 (100%)	
Yes	670 (25%)	0 (0%)	149 (40%)	300 (38%)	221 (24%)	0 (0%)	

SIMD: Scottish Index of Multiple Deprivation; ASA: American Society of Anesthesiologists Grade; EMVI: extramural venous invasion; SIG: systemic inflammatory grade.

*Chi-squared test.

**Table 3:** Association between age and CT-derived body composition.

	Total	< 50	50s	60s	70s	80+	p*
Males only							
BMI	980	44 (5%)	151 (15%)	318 (32%)	338 (35%)	129 (13%)	< 0.001
< 18.5	16 (2%)	4 (9%)	3 (2%)	3 (1%)	3 (1%)	3 (2%)	
18.5–24.9	278 (28%)	16 (36%)	36 (24%)	72 (23%)	102 (30%)	52 (40%)	
25–29.9	392 (40%)	15 (34%)	61 (40%)	124 (39%)	143 (42%)	49 (38%)	
30–34.9	208 (21%)	6 (14%)	30 (20%)	82 (26%)	66 (20%)	24 (19%)	
35+	86 (9%)	3 (7%)	21 (14%)	37 (12%)	24 (7%)	1 (1%)	
SFI	1189	45 (4%)	167 (14%)	388 (33%)	414 (35%)	175 (15%)	< 0.001
Normal	363 (31%)	19 (42%)	48 (29%)	106 (27%)	112 (27%)	78 (45%)	
High	826 (70%)	26 (58%)	119 (71%)	282 (73%)	302 (73%)	97 (55%)	
Visceral obesity	1326	50 (4%)	177 (13%)	425 (32%)	461 (35%)	213 (16%)	< 0.001
Normal	297 (22%)	30 (60%)	42 (24%)	80 (19%)	81 (18%)	64 (30%)	
High	1029 (78%)	20 (40%)	135 (76%)	345 (81%)	380 (82%)	149 (70%)	
SMI	926	42 (5%)	143 (15%)	305 (33%)	318 (34%)	118 (13%)	< 0.001
Normal	452 (49%)	28 (67%)	96 (67%)	179 (59%)	116 (37%)	33 (28%)	
Low	474 (51%)	14 (33%)	47 (33%)	126 (41%)	202 (64%)	85 (72%)	
SMD	928	42 (5%)	143 (15%)	305 (33%)	318 (34%)	120 (13%)	< 0.001
Normal	315 (34%)	33 (79%)	80 (56%)	123 (40%)	67 (21%)	12 (10%)	
Low	613 (66%)	9 (21%)	63 (44%)	182 (60%)	251 (79%)	108 (90%)	
Females only							
BMI	900	62 (7%)	146 (16%)	276 (31%)	296 (33%)	120 (13%)	< 0.001
< 18.5	25 (3%)	3 (5%)	3 (2%)	8 (3%)	8 (3%)	3 (3%)	
18.5–24.9	341 (38%)	28 (45%)	45 (31%)	98 (36%)	108 (37%)	62 (52%)	
25–29.9	266 (30%)	16 (26%)	47 (32%)	88 (32%)	82 (28%)	33 (28%)	
30–34.9	155 (17%)	6 (10%)	19 (13%)	45 (16%)	67 (23%)	18 (15%)	
35+	113 (13%)	9 (15%)	32 (22%)	37 (13%)	31 (11%)	4 (3%)	
SFI	1126	64 (6%)	169 (15%)	312 (28%)	377 (34%)	204 (18%)	0.023
Normal	108 (10%)	5 (8%)	9 (5%)	29 (9%)	34 (9%)	31 (15%)	
High	1018 (90%)	59 (92%)	160 (95%)	283 (91%)	343 (91%)	173 (85%)	
Visceral obesity	1249	67 (5%)	174 (14%)	333 (27%)	422 (34%)	253 (20%)	0.006
Normal	321 (26%)	24 (36%)	44 (25%)	69 (21%)	102 (24%)	82 (32%)	
High	928 (74%)	43 (64%)	130 (75%)	264 (79%)	320 (76%)	171 (68%)	
SMI	861	59 (7%)	140 (16%)	266 (31%)	280 (33%)	116 (14%)	< 0.001
Normal	367 (43%)	34 (58%)	79 (56%)	117 (44%)	109 (39%)	28 (24%)	
Low	494 (57%)	25 (42%)	61 (44%)	149 (56%)	171 (61%)	88 (76%)	
SMD	864	59 (7%)	140 (16%)	266 (31%)	281 (33%)	118 (14%)	< 0.001
Normal	241 (28%)	41 (70%)	68 (49%)	85 (32%)	41 (15%)	6 (5%)	
Low	623 (72%)	18 (31%)	72 (51%)	181 (69%)	240 (85%)	112 (95%)	

BMI: body mass index; SFI: subcutaneous fat index; SMI: skeletal muscle index; SMD: skeletal muscle density.

*Chi squared test.

### Association between age and clinicopathological factors

The association between age and tumour/host factors is shown in Table 2. Increasing age was associated with increased ASA grade (p < 0.001), an increased proportion of right sided tumours (p < 0.001) and node negative disease (p < 0.001). Extremes of age (< 50 or 80+) were associated with female sex (p = 0.007), emergency presentation (p < 0.001), poorly differentiated tumours (p = 0.007), extramural venous invasion (p = 0.017) and increased systemic inflammatory grade (p < 0.001). No significant association was seen between age at presentation and socioeconomic deprivation (p = 0.091). 0%/40%/38%/24%/0% of cancers were diagnosed thorough screening within the < 50/50s/60s/70s/80+ age groups respectively.

**Figure 2:**
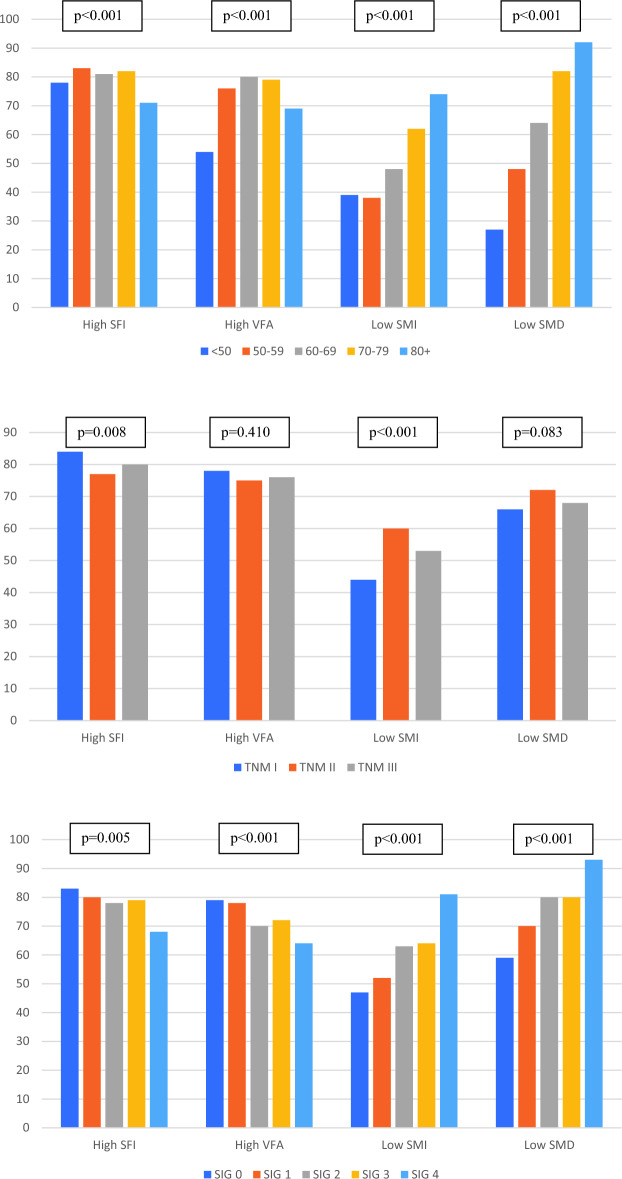
Association between proportion of patients with high SMI, high VFA, low SMI and: Top—age at diagnosis. Middle—TNM stage. Bottom—systemic inflammatory grade

The association between age and anthropometric measurements is shown in Table 3. Patients at extremes of age were more likely to have a BMI < 25 (p < 0.001) and a normal subcutaneous fat index and visceral fat area (all p < 0.001). Increasing age was associated with low skeletal muscle index and low skeletal muscle density (39%/38%/48%/62%/74% and 27%/48%/64%/82%/92% for age < 50/50–59/60–69/70–79/80+ respectively (both p < 0.001)). When this analysis was repeated in male and female patients separately similar results were observed as shown in Table 3. The association between CT-derived body composition, age, TNM stage and systemic inflammatory grade is shown graphically in Fig. 2.

### Association between subcutaneous fat index and tumour/host factors

The association between subcutaneous fat index and tumour/host factors is shown in Table 4.

**Table 4:** Association between SFI/VFA and tumour and host factors (uni- and multi-variate analysis).

	SFI (normal vs high)				VFA (normal vs high)			
	UVA		MVA		UVA		MVA	
	OR (95% CI)	p	OR (95% CI)	p	OR (95% CI)	p	OR (95% CI)	p
Age	0.88 (0.79–0.96)	0.006	–	0.448	1.03 (0.94–1.12)	0.542	–	–
Sex	4.14 (3.28–5.23)	< 0.001	7.09 (4.70–10.69)	< 0.001	0.83 (0.70–1.00)	0.050	–	0.449
SIMD	0.94 (0.88–1.01)	0.102	–	–	0.94 (0.88–1.00)	0.055	–	0.971
ASA	0.96 (0.82–1.11)	0.554	–	–	1.14 (1.00–1.30)	0.058	1.30 (1.01–1.69)	0.044
Mode of press	0.55 (0.42–0.72)	< 0.001	–	0.070	0.48 (0.39–0.61)	< 0.001	0.65 (0.43–0.97)	0.036
BMI	4.84 (3.95–5.93)	< 0.001	6.60 (4.84–9.01)	< 0.001	7.97 (6.30–10.07)	< 0.001	7.19 (5.32–9.73)	< 0.001
Tumour side	0.92 (0.75–1.13)	0.435	–	–	1.20 (1.00–1.44)	0.056	–	0.404
TNM stage	0.94 (0.82–1.08)	0.353	–	–	0.94 (0.83–1.07)	0.352	–	–
Diff	0.98 (0.75–1.27)	0.861	–	–	0.73 (0.58–0.91)	0.006	–	0.775
EMVI	0.75 (0.61–0.93)	0.007	–	0.214	0.80 (0.67–0.96)	0.018	–	0.544
SIG	0.85 (0.77–0.93)	< 0.001	–	0.996	0.84 (0.77–0.91)	< 0.001	–	0.638

Categories: age (50s*/60s/70s/80+), sex (male*/female), SIMD (1*/2/3/4/5), ASA (1*/2/3/4), mode of presentation (elective*/emergency), BMI (< 18.5*/18.5–24.9/25–29.9/30–34.9/35+), tumour side (right*/left), TNM stage (1*/2/3), differentiation (well-mod*/poorly differentiated), EMVI (negative*/positive), SIG (0*/1/2/3/4).

UVA: univariate analysis; MVA: multivariate analysis; OR: odds ratio; CI: confidence interval; SIMD: Scottish Index of Multiple Deprivation; ASA: American Society of Anesthesiologists Grade; BMI: body mass index; Diff: differentiation; EMVI: extramural venous invasion; SIG: systemic inflammatory grade.

On univariate analysis: age (p = 0.006), sex (p < 0.001), mode of presentation (p < 0.001), BMI (p < 0.001), EMVI (p = 0.007) and SIG (p < 0.001) were associated with subcutaneous fat index. No association was seen between subcutaneous fat index and SIMD (p = 0.102), ASA grade (p = 0.554), tumour side (p = 0.435) or differentiation (p = 0.861).

When those factors significant (p < 0.01) on univariate analysis were entered into the multivariate model female sex (OR 7.09, p < 0.001) and higher BMI (OR 6.60, p < 0.001) remained independently associated with high subcutaneous fat index.

### Association between visceral fat area and tumour/host factors

The association between visceral fat area and tumour/host factors is shown in Table 4. On univariate analysis: sex (p = 0.050), SIMD (p = 0.055), ASA (p = 0.058), mode of presentation (p < 0.001), BMI (< 0.001), tumour side (p = 0.056), differentiation (p = 0.006), EMVI (p = 0.018) and SIG (p < 0.001) were associated with visceral fat area. No association was seen between visceral fat area and age (p = 0.542) or TNM Stage (p = 0.352).

When those factors significant (p < 0.01) on univariate analysis were entered into the multivariate model: higher ASA (OR 1.30, p = 0.044), elective presentation (OR 0.65, p = 0.036) and BMI (OR 7.19, p < 0.001) were associated with high visceral fat area.

### Association between skeletal muscle index and tumour/host factors

The association between skeletal muscle index and tumour/host factors is shown in Table 5. On univariate analysis: age (p < 0.001), sex (p = 0.009), SIMD (p < 0.001), ASA (p < 0.001), mode of presentation (p < 0.001), BMI (p < 0.001), tumour side (p = 0.055), EMVI (p = 0.007) and SIG (p < 0.001) were associated with skeletal muscle index. No association was seen between skeletal muscle index and TNM Stage (p = 0.114) or differentiation (p = 0,719).

**Table 5:** Association between SMI/SMD and tumour and host factors (uni- and multi-variate analysis).

	SMI				SMD			
	UVA		MVA		UVA		MVA	
	OR (95% CI)	p	OR (95% CI)	p	OR (95% CI)	p	OR (95% CI)	p
Age	1.57 (1.43–1.72)	< 0.001	1.47 (1.29–1.68)	< 0.001	2.32 (2.08–2.59)	< 0.001	2.28 (1.94–2.67)	< 0.001
Sex	1.28 (1.07–1.55)	0.009	1.34 (1.03–1.76)	0.032	1.33 (1.09–1.63)	0.006	1.38 (1.02–1.87)	0.038
SIMD	1.11 (1.05–1.19)	< 0.001	1.15 (1.05–1.26)	0.004	0.94 (0.88–1.01)	0.080	–	0.970
ASA	1.51 (1.30–1.75)	< 0.001	1.30 (1.04–1.63)	0.019	2.61 (2.19–3.11)	< 0.001	1.92 (1.49–2.48)	< 0.001
Mode of press	2.09 (1.55–2.81)	< 0.001	1.82 (1.23–2.69)	0.003	1.75 (1.26–2.43)	< 0.001	1.71 (1.08–2.71)	0.023
BMI	0.58 (0.53–0.64)	< 0.001	0.67 (0.59–0.77)	< 0.001	0.93 (0.84–1.02)	0.127	–	–
Tumour side	0.83 (0.69–1.00)	0.055	–	0.929	0.77 (0.63–0.95)	0.013	–	0.095
TNM stage	1.11 (0.98–1.26)	0.114	–	–	1.00 (0.87–1.14)	0.979	–	–
Diff	1.05 (0.82–1.35)	0.719	–	–	1.02 (0.78–1.34)	0.889	–	–
EMVI	1.30 (1.08–1.57)	0.007	–	0.344	1.10 (0.90–1.35)	0.359	–	–
SIG	1.38 (1.25–1.52)	< 0.001	1.23 (1.10–1.38)	< 0.001	1.41 (1.26–1.57)	< 0.001	1.37 (1.19–1.58)	< 0.001

Categories: age (50s*/60s/70s/80+), sex (male*/female), SIMD (1*/2/3/4/5), ASA (1*/2/3/4), mode of presentation (elective*/emergency), BMI (< 18.5*/18.5–24.9/25–29.9/30–34.9/35+), tumour side (right*/left), TNM stage (1*/2/3), differentiation (well-mod*/poorly differentiated), EMVI (negative*/positive), SIG (0*/1/2/3/4).

UVA: univariate analysis; MVA: multivariate analysis; OR: odds ratio; CI: confidence interval; SIMD: Scottish Index of Multiple Deprivation; ASA: American Society of Anesthesiologists Grade; BMI: body mass index; Diff: differentiation; EMVI: extramural venous invasion; SIG: systemic inflammatory grade.

When those factors significant (p < 0.01) on univariate analysis were entered into the multivariate model: older age (OR 1.47, p < 0.001), female sex (OR 1.34, p = 0.032), less deprivation (OR 1.15, p = 0.004), higher ASA (OR 1.30, p = 0.019), emergency presentation (OR 1.82, p < 0.001), lower BMI (OR 0.67, p < 0.001) and higher SIG (OR 1.23, p < 0.001) were associated with low skeletal muscle index.

### Association between skeletal muscle density and tumour/host factors

The association between skeletal muscle density and tumour/host factors is shown in Table 5. On univariate analysis: age (p < 0.001), sex (p = 0.006), SIMD (p = 0.080), ASA (p < 0.001), mode of presentation (p < 0.001), tumour side (p = 0.013) and SIG (p < 0.001) were associated with skeletal muscle density. No association was seen between skeletal muscle density and BMI (p = 0.127), TNM Stage (p = 0.979), differentiation (p = 0.889) or EMVI (p = 0.359).

When those factors significant (p < 0.01) on univariate analysis were entered into the multivariate model: age (OR 2.28, p < 0.001), female sex (OR 1.38, p = 0.038), higher ASA (OR 1.92, p < 0.001), emergency presentation (OR 1.71, p = 0.023) and higher SIG (OR 1.37, p < 0.001) were associated with low skeletal muscle density.

## Discussion

The results of the present study show that within a large cohort of patients undergoing resectional surgery with curative intent for TNM I-III colon cancer, abnormal CT-derived body composition, in particular low skeletal muscle mass and density, is highly prevalent. Increasing age was the major factor associated with low skeletal muscle mass and density although an additional association with the systemic inflammatory response and (to a lesser extent) TNM Stage was observed. Therefore, it would appear likely that low skeletal muscle mass/density is largely constitutional as opposed to being a disease mediated effect.

CT and MRI quantification of muscle mass are now widely accepted to be gold standard however this was traditionally evaluated by other methods including dual energy x-ray absorptiometry (DXA), bioimpedance analysis and skinfold anthropometry^34^. Given the relative paucity of routinely collected data for muscle function; the terms sarcopenia and low skeletal muscle index/mass are often used interchangeably. A significant association has been reported between muscle density and function^35^ therefore skeletal muscle density may provide a radiological estimate of muscle function. To date, there is no standardized methodology for stratification of body composition within the published literature, both in terms of Hounsfield unit ranges for subcutaneous fat, visceral fat and skeletal muscle and the methodology used for stratifying normal/abnormal. As recently recommended by Klassen and colleagues^36^, established methodology would be useful particularly when analysing change in specific populations and comparing populations. The present study utilized the most widely published methods of stratifying body composition from CT imaging.

A previous review reported a prevalence of sarcopenia of 24–40% based on muscle mass and 10–19% based on muscle mass in addition to strength/function within adults aged over 60 years^37^. However, the included studies did not utilize cross sectional imaging and the present results would suggest that when quantified using gold standard imaging techniques, the incidence of low skeletal muscle mass in older patients may be significantly higher than previously reported^38^.

Low skeletal muscle mass and density is recognized to be prevalent in patients with cancer and associated with adverse short-term/long-term outcomes and increased toxicity to chemotherapy^12,39,40^. Reduced muscle mass now represents one of the phenotypic criteria for the diagnosis of malnutrition as defined within the GLIM criteria, typically as a result of reduced food intake/absorption or increased disease burden or inflammation. However, more recently, studies have suggested that the effect of the tumour (in terms of tumour stage) on body composition is minimal however an association has been reported with the systemic inflammatory response^22^. The results of the present study show that despite there being an evident association between lean muscle mass and the systemic inflammatory response, older age markedly outweighs the effect of either the tumour or the systemic inflammatory response on skeletal muscle mass or density. Although validated methods of CT-derived body composition analysis adjust for sex, based on the present results it would appear likely that in order to examine the effect of the underlying disease process on body composition requires adjustment for age. A previous study by Magudia and colleagues^41^ did report an association between age and body composition in patients without malignancy or cardiovascular disease. However, this study included scans carried out as diagnostic investigations (indications not clearly defined) therefore cannot be assumed to be a normal healthy population. Furthermore, it did not analyse the association with subcutaneous fat index, visceral fat area or skeletal muscle density. A comparison of CT-derived body composition after adjustment for age would be of interest between patients with cancer and the normal, healthy population. Due to the radiation exposure of CT imaging this is limited by ethical constraints. Perhaps it may be possible to identify a cohort of patients without malignant disease where an impact on CT derived body composition would not be anticipated however other previously studied conditions including cardiovascular disease have an association with the systemic inflammatory response and therefore could not truly be considered a normal healthy control group.

Within colorectal cancer, age-specific incidence rates are highest within the 85 to 89 age group therefore the present findings carry significant implications^42^. To date, the dosing of chemotherapeutic agents has been largely based on body surface area (BSA) or body mass index (BMI). Body surface area is recognized to be poorly associated with lean muscle mass^43^ and previous studies including a systematic review have reported an association between sarcopenia and chemotoxicity^15,43–45^. Dose modification is common and been reported to be required in 20–62% of patients within colorectal cancer trials^46^. Elderly patients are recognized to have a higher rate of chemotoxicity and furthermore, are typically underrepresented within clinical trials^46,47^. A previous study^48^ of 318 patients aged 80 years and older reported high levels of dose reduction (41%), therapy discontinuation (32%) and hospitalization (32%). Patients may have suboptimal outcomes due to overdosing resulting in chemotoxic side effects or conversely underdosing due to early cessation of chemotherapy due to these adverse effects. Given the high proportion of low skeletal muscle mass/density within the older population coupled with the previously reported findings as described above, the routine evaluation of skeletal muscle mass/density for dosing of chemotherapy may improve outcomes, particularly within the older population. Further investigation of this would be of interest in future studies. Conversely patients, typically younger, with a higher lean muscle mass may tolerate higher chemotherapy dosing than is currently administered with potentially improved outcomes.

Aside from body composition, a number of other factors were associated with patient age. The association between age: tumour stage, extramural venous invasion (EMVI) and mode of presentation is likely to be related to the routine invitation of people aged 50–74 to participate in the bowel screening programme. The association between increasing comorbidity (as measured by ASA) and advancing age is unsurprising. The association between advancing age and increasing proportion of right sided colon cancer is consistent with previous literature^49–52^. Although a number of possible explanations have been hypothesized the reason for this observation remains uncertain^51^.

Due to the retrospective nature of this study there was missing data, particularly in terms of body mass index and preoperative C-reactive protein. Nonetheless, this was a large study and the effect of this missing data is likely to be minimal. In keeping with the majority of the literature, total body composition has been estimated from a single imaging slice at the level of the third lumbar vertebrae. This method has been previously shown to be a reliable estimation of both skeletal muscle and adipose issue volume^5,30^ and is felt unlikely to be a significant limitation. CT imaging is typically used within the literature instead of MRI as it is routinely performed in patients with colon cancer and has been shown to be highly correlated with MRI based findings^53^. A recent review reported that several CT imaging related factors including the effect of CT scan kilovoltage and the use of contrast have a significant effect on muscle analysis. In the present study contrast scans in the portal venous phase were analysed however we were unable to account for the kilovoltage administered. Nonetheless, this is a novel finding that is not routinely considered in body composition analysis however could be considered in future studies^54^. This study utilised a large cohort of patients from the West of Scotland. This population is felt to be representative of the whole of Scotland and likely representative of similar Western nations. However, the findings of this study may not be applicable to non-Caucasian or eastern populations and further studies within non-Caucasian populations are required. There may be other clinicopathological factors not accounted for in the present study that may influence body composition. There may include lifestyle factors including diet^55^ and exercise^56^ that could not be accounted for on an individual level within this study as the relevant data was not available. Socioeconomic deprivation has been included and this is a surrogate for a number of factors including diet and exercise. However, further research that includes lifestyle factors on an individual level would be of interest. Other factors including the gut microbiome^57^ and medication^58^ may be of interest in future research however it seems likely that age is the predominant factor relating to skeletal muscle mass and density with the systemic inflammatory response playing an additional role. It may be that some of these other factors may influence muscle mass and density through manipulation of the systemic inflammatory response.

In conclusion, the present results show the association between age and CT-derived body composition within a cohort of patients with colon cancer. In order to determine the disease effect on body composition it would appear likely that stratification for age is required. Furthermore, the present results in addition to previous literature suggest a possible need for consideration of evaluation of lean muscle mass using gold-standard techniques to reduce chemotherapy toxicity, particularly within older patients.

## Data Availability

Data is available on request. The corresponding author (Allan Golder) should be contacted for such requests.
